# GM1 and GM2-Gangliosidosis: Clinical Features, Neuroimaging Findings and Electroencephalography

**DOI:** 10.22037/ijcn.v18i2.40751

**Published:** 2024-03-12

**Authors:** Parvaneh KARIMZADEH, Masomeh EBRAHIMI, Korosh ETEMAD, Farzad AHMAD ABADI, Zahra HOSSEINI NEZHAD

**Affiliations:** 1 Pediatric Neurology Research Center, Shahid Beheshti University of Medical Sciences, Tehran, Iran.; 2 Pediatric Neurology Department, Mofid Children’s Hospital, Faculty of Medicine, Shahid Beheshti University of Medical Sciences, Tehran, Iran; 3 Department of Epidemiology, Faculty of Medicine, Shahid Beheshti University of Medical Sciences, Tehran, Iran.; 4 Department of Pediatrics, Faculty of Medicine, Baqiyatallah University of Medical Sciences, Tehran, Iran.

**Keywords:** GM1 & GM2-Gangliosidosis, Developmental Delay, Neurometabolic Disorders, Genetic Disorders

## Abstract

**Abstract:**

Gangliosidosis is one of the hereditary metabolic diseases caused by the accumulation of Gangliosid in the central nervous system, leading to severe and progressive neurological deficits. Regarding phenotype, GM1 and GM2-Gangliosidosis are divided into Infantile, Juvenile, and Adult.

**Materials & Methods:**

In this study, thirty-seven patients with GM1 and GM2-Gangliosidosis were referred to the neurology department of Mofid Children’s Hospital in Tehran, Iran, whose disease was confirmed from September 2019 to December 2021. This study assessed age, sex, and developmental status before the onset of the disease, clinical manifestations, brain imaging, and electroencephalography.

**Results:**

97.20% of patients were the result of family marriage. Approximately 80% of juvenile patients were developmentally normal before the onset of the disease. Developmental delay was more common among infantile GM1-Gangliosidosis than infantile GM2-Gangliosidosis, but in total, more than 50% of GM1&GM2-Gangliosidosis patients had reached their developmental milestone before the onset of the disease. With the onset of disease symptoms, 100% of patients regressed in terms of movement, 97.20% of them mentally, and 75% of them had seizures during the disease. The most common clinical findings were cherry-red spot, Mongolian spot, macrocephaly, organomegaly, hyperacusis, and scoliosis. The most common brain imaging findings included bilateral thalamus involvement, brain atrophy, PVL, and delayed myelination. The most common finding in electroencephalography was background low voltage with abnormal sharp waves.

**Conclusion:**

This study concluded that most of the patients are the result of family marriage, and most of the juvenile patients are developmentally normal before the onset of the disease. In addition, more than 50% of infantile patients reach their developmental milestones before the onset of the disease. The most common clinical findings of these patients are seizures, cherry-red spot, macrocephaly, hyperacusis, Mongolian spot, and bilateral involvement of the thalamus.

## Introduction

Gangliosidosis is an inherited metabolic disorder caused by the accumulation of Gangliosides in the central nervous system, which leads to severe and progressive neurological disorders. They are classified as GM1-Gangliosidosis and GM2-Gangliosidosis, both autosomal recessive diseases. GM1-Gangliosidosis (OMIM #230500) is caused by a mutation in the GLB1 gene, resulting in a deficiency of the lysosomal enzyme β-Galactosidase and the subsequent accumulation of GM1-Gangliosidosis. 

GM2-Gangliosidosis, including Thy-Sachs disease (OMIM #272800) and Sandhoff disease (OMIM #268800), are caused by mutations in the HEXA and HEXB genes, which encode the α and β subunits of the enzyme lysosomal β-hexosaminidase, respectively, leading to Accumulation of GM2-Gangliosidosis(1).

GM1 and GM2-Gangliosidosis phenotypes are generally classified as early infantile, late infantile, juvenile, and adult. Patients with the infantile form show signs of the disease during infantile, presenting with progressive neurological disorders and death in early childhood (2,3). A late infantile has also been reported in which patients show symptoms between one and three years of age (3,4). The onset of symptoms in the juvenile form is usually between three and five years of age, manifesting as ataxia, dysarthria, hypotonia, and dysphagia (5,6). The life span of adolescents varies from late childhood to early adulthood (1). In contrast, the adult form (or late onset) presents symptoms in early or mid-adulthood. The symptoms include pelvic girdle weakness, ataxia, neuromuscular weakness, and eventually loss of independent movement ability (7,8). In addition, speech problems may occur. Patients may also experience psychological changes (3,5,6). The lifespan of the adult form varies considerably (6). No effective treatment exists for GM1and GM2-Gangliosidosis, and palliative measures are the current standard of care. However, many ongoing efforts exists to develop therapeutic protocols, including the development of new animal models (9), enzyme replacement therapy (10), substrate reduction therapy (11), bone marrow transplantation (12), and gene therapy in animal models (13). This study presents the clinical findings of thirty-seven patients with GM1&GM2-Gangliosidosis were referred to the neurology department of Mofid Children’s Hospital of Shahid Beheshti University of Medical Sciences in Tehran, Iran.

## Materials & Methods

This study analyzed the clinical findings of thirty-seven patients with GM1and GM2-Gangliosidosis. These patients were referred to the neurology department of Mofid Children’s Hospital of Shahid Beheshti University of Medical Sciences (SBUMS) between 2019 and 2021, and their disease was confirmed with genetic tests, and hexosaminidase enzyme and siblings confirmed patients with genetic or enzyme tests whose clinical symptoms were consistent with GM1 and GM2-Gangliosidosis (two patients). the researchers collected the demographic information of the patients, including age, sex and type of disease, development status of the patients based on the questionnaire designed from the ASQ3TM test before the onset of the disease symptoms retrospectively, the clinical symptoms of the patients and the findings of their imaging and electroencephalography, and we did a descriptive study without statistical analysis.

Institutional ethical approval for conducting this study was obtained from the Pediatric Neurology Research Center of SBUMS. All parents signed a written consent for participation in this study.

## Results

Of the thirty-seven patients in this study, twenty were boys (60%), and 17 were girls (40%).

The age range of patients participating in this research was 0.9 to 14.2 years (average 4.027 years).

Twelve patients were GM1-Gangliosidosis consisting of one case early infantile, ten cases late infantile, and one case juvenile. Tweny-five patients were GM2-gangliosidosis consisting of 12 cases Tay-Sachs (infantile), five cases Tay-Sachs (juvenile, adult), five cases Sandhoff (infantile) and three cases Sandhoff (juvenile, adult).

Thirty-six patients (97.2%) were the result of family marriage, and only one patient had unrelated parents.

The disease of twenty-eight people (75.6%) out of thirty-seven studied patients was confirmed by genetic test (40.5% of patients with genetic test and hexoseaminidase enzyme and 35.1% of patients only with genetic test), and the disease of 7 patients (18.9%) was confirmed only by checking hexosaminidase enzyme. Two patients were included in the study according to the clinical symptoms corresponding to GM1 and GM2-Gangliosidosis and the confirmation of their sibling’s disease by genetic and enzyme tests (one genetic case and one enzyme case). All patients had symptoms consistent with GM1 & GM2-Gangliosidosis.

The development of the patients before the onset of the clinical symptoms of the disease was as follows: In GM1-Gangliosidosis, five of the 12 studied patients (41.6%) were normal in Gross Motor (one of them juvenile, and 4(36%) of them infantile) and the remaining seven patients in this group (58.4%) had some degree of developmental delay (all of them infantile (64%)). 

In GM2-Gangliosidosis, 14 of the twenty-five studied patients (56%) were normal in Gross Motor 7(87%) of them juvenile and 7(41%) of them infantile, and the remaining 11 patients in this group (44%) had some degree of developmental delay, one of them (13%) juvenile and 10 (59%) of them infantile.

Moreover, the developmental status of patients in fine motor before the onset of clinical symptoms of the disease in 12 GM1-Gangliosidosis, five patients (41.6%) were normal, one of them juvenile, and 4(36%) of them infantile, and the rest seven patients of this group (58.3%) had some degree of developmental delay, all of them infantile (64%).

In GM2-Gangliosidosis, 18 of the twenty-five patients (72%) had normal fine motor, 8 (100%) of them were juvenile, and 10 (59%) of them were infantile; the remaining seven patients of this group (28%) had some degree of developmental delay, all of them infantile (41%).

Developmental status of patients in communication and language before the onset of clinical symptoms of the disease: in the 12 GM1-Gangliosidosis, four patients (33.3%) were normal, all of them were infantile (36%), and the remaining eight patients of this group (66.7%) had some degree of developmental delay, one of them juvenile and 7(64%) of them were infantile.

In the GM2-Gangliosidosis, 19 of the 25 patients (76%) were normal in communication and language, 8 (100%) of them were juvenile, 11(65%) of them were infantile, and the remaining six patients in this group (24%) had some degree of developmental delay, all of them were infantile (35%).

Developmental status of patients in Social Skills before the onset of clinical symptoms: in 12 GM1- Gangliosidosis, five patients (41.6%) were normal, one of them was juvenile, 4 (36%) of them were infantile, and the remaining seven patients of this group (58.4%) had some degree of developmental delay, all of them were infantile(64%).

In the GM2-Gangliosidosis group, 19 of the 25 patients (76%) were normal, (8 (100%) of them were juvenile, 11(65%) of them were infantile), and the remaining six patients in this group (24%) had some degree of developmental delay, all of them were infantile (35%).

Notably, only one juvenile patient of GM1-Gangliosidosis was delayed in communication and language, one juvenile patient out of eight patients of GM2-Gangliosidosis was delayed in the Gross Motor, and in other areas, all these patients were normal (Table 1).

The clinical symptoms of thirty-seven patients in this study were as follows: 

Fifteen of thirty-seven patients (40.5%) had organomegaly. One early infantile and four late infantile of GM1-Gangliosidosis had hepatomegaly and two late infantile also had hepatosplenomegaly. Eight patients of GM2-Gangliosidosis had hepatomegaly.

All 12 patients of GM1-Gangliosidosis experienced motor and mental regression during the disease. All twenty-five patients with GM2-Gangliosidosis had motor regression, and twenty-four patients had mental regression.

Twenty-eight patients (75.7%) studied had seizures during the disease, and only 24.3% (9 patients) had not yet experienced seizures.

Most patients (64.3%) had mixed-type seizures, and eight patients (14.3%) had generalized tonic-colonic seizures.

In the GM1-Gangliosidosis group, two patients of late infantile had hyperacusis. One early infantile and seven late infantile had cherry red spot. Five late infantile had macrocephaly, one early infantile, seven late infantile had Mongolian spot, and three late infantile had scoliosis.

From the twenty-five patients of GM2-Gangliosidosis, nine patients of Tay-Sachs

**Table 1:** Developmental status of GM1 & GM2-Gangliosidosis patients before the onset of clinical symptoms of the disease

		Gross Motor		Fine Motor		Communication & Language		Social Skills		Total
		Normal	Abnormal	Normal	abnormal	Normal	abnormal	Normal	Abnormal	
GM1	Early	-	1	-	1	-	1	-	1	1
	Late	4	6	4	6	4	6	4	6	10
	Juvenile	1	-	1	-	-	1	1	-	1
	Total	5(41.6%)	7(58.4%)	5(41.6%)	7(58.4%)	4(33.3%)	8(66.7%)	5(41.6%)	7(58.4%)	12(100%)
GM2	Tay-Sachs (infantile)	5	7	8	4	9	3	9	3	12
	Tay-Sachs (juvenile, adult)	5	-	5	-	5	-	5	-	5
	Sandhoff (infantile)	2	3	2	3	2	3	2	3	5
	Sandhoff (juvenile, adult)	2	1	3	-	3	-	3	-	3
	Total	14(56%)	11(44%)	18(72%)	7(28%)	19(76%)	6(24%)	19(76%)	6(24%)	25(100%)
Grand total		19	18	23	14	23	14	23	14	37
%		51.4%	48.6%	62.2%	37.8%	62.2%	37.8%	62.2%	37.8%	100%

**Table 2:** Clinical findings of GM1 & GM2-Gangliosidosis patients

		organomegaly		regression		seizure							
		Hepatomegaly	Hepato – splenomegaly	Mental	Motor	Yes	No	Hyperacusis	C.R.S	Macrocephal	Mongolian spot	Scoliosis	Total
GM1	Early	1	-	1	1	-	1	-	1	-	1	-	1
	Late	4	2	10	10	8	2	2	7	5	7	3	10
	Juvenile	-	-	1	1	-	1	-	-	-	-	-	1
	Total	5	2	12	12	8	4	2	8	5	8	3	12
GM2	Tay-sachs (infantile)	5	-	12	12	9	3	9	12	12	4	3	12
	Tay-sachs (juvenile, adult)	1	-	5	5	4	1	2	-	-	-	-	5
	Sandhoff (infantile)	1	-	5	5	5	-	3	5	1	1	-	5
	Sandhoff (juvenile, adult)	1	-	2	3	2	1	-	-	-	-	-	3
	Total	8	-	24	25	20	5	14	17	13	5	3	25
Grand total		13	2	36	37	28	9	16	25	18	13	6	37
%		35.1%	5.4%	97.3%	100.0%	75.7%	24.3%	43.2%	67.6%	48.6%	35.1%	16.2%	100%

**Table 3:** Brain MRI findings of GM1 & GM2-Gangliosidosis patients

		Normal	Diffuse cerebellar atrophy	PVL	Diffuse brain atrophy & bilateral thalamus involvement	Bilateral thalamus involvement	Diffuse brain atrophy	Subdural space in fronto-parietal	Extensive white matter signal change	Delayed myelination	Mild ventricolomegaly	Hyper intensity in BG in T2 (lentiform nucleus)	Total
GM1	Early	1	-	-	-	-	-	-	-	-	-	-	1
	Late	1	-	-	-	2	-	-	-	-	1	1	10
	Juvenile	-	-	1	-	-	-	-	-	-	-	-	1
	Total	2	-	1	-	2	-	-	-	-	1	1	12
GM2	Tay-sachs (infantile)	2	-	1	1	2	1	-	-	1	-	-	12
	Tay-sachs (juvenile, adult)	2	-	-	-	-	-	-	-	-	-	-	5
	Sandhoff (infantile)	-	-	-	-	1	-	1	1	1	-	-	5
	Sandhoff (juvenile, adult)	-	1	1	-	-	1	-	-	-	-	-	3
	Total	4	1	2	1	3	2	1	1	2	-	-	25
Grand total		6	1	3	1	5	2	1	1	2	1	1	37

**Fig 1:**
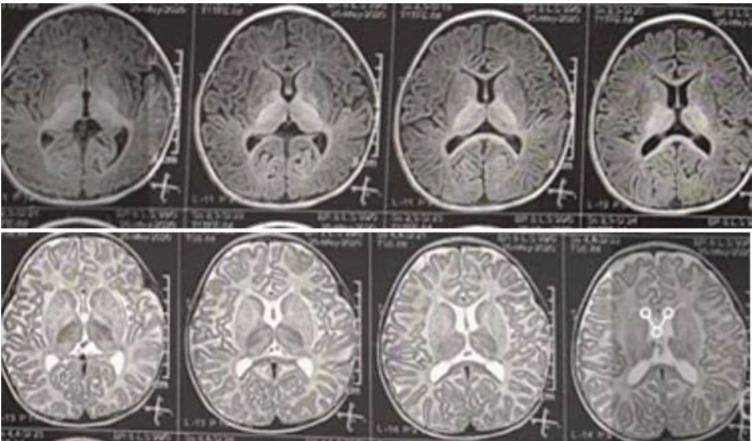
A 15-month-old female patient with late GM1-Gangliosidosis with bilateral thalamic involvement in T1 and T2 sequence of the brain MRI

**Fig 2:**
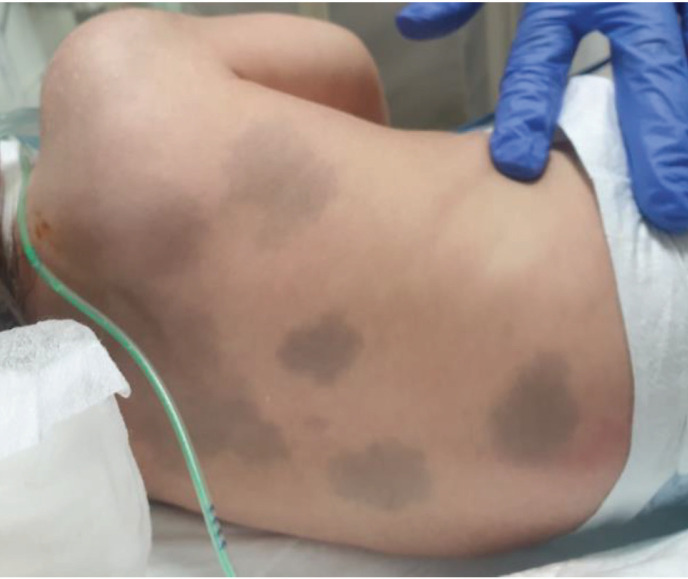
A 15-month-old female patient with late GM1-Gangliosidosis characterized with Mongolian spot in the skin

**Fig 3:**
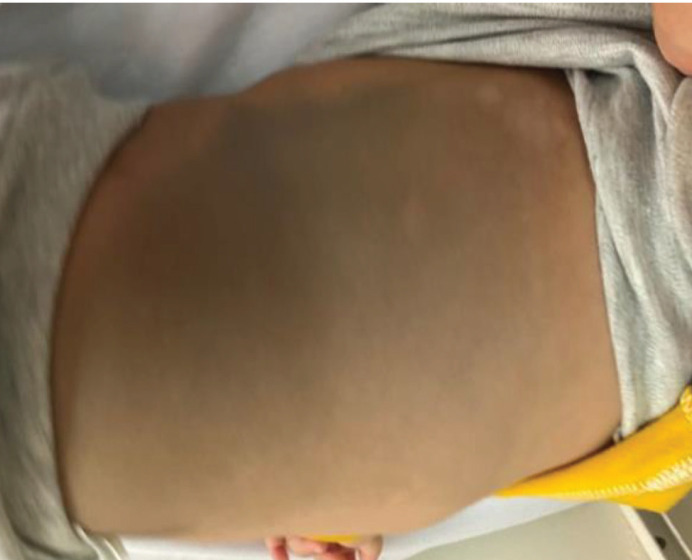
A 10-month-old male patient with late (Tay-sachs) GM2 -Gangliosidosis characterized with Mongolian spot in the skin

**Fig 4:**
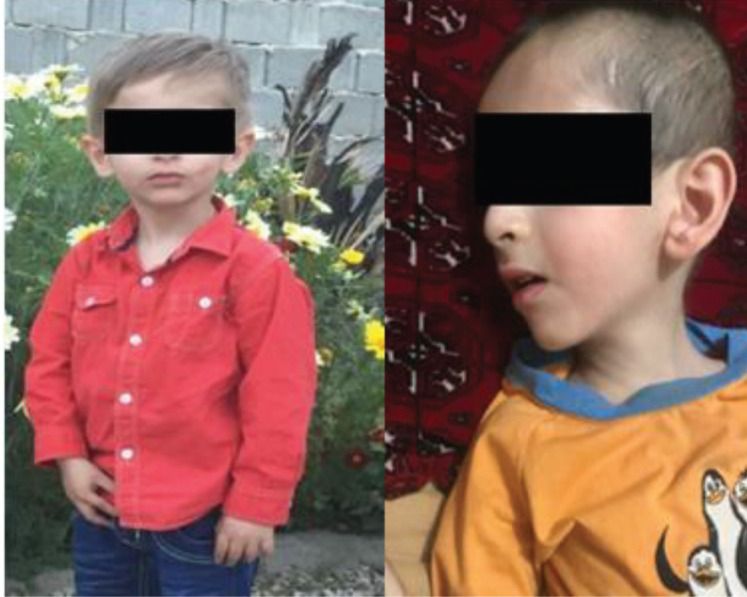
A 3-year-old male patient with juvenile (Tay-sachs) GM2-Gangliosidosis with normal development before the onset of the disease and 2 years after the onset of the disease

## Discussion

Gangliosidosis is an inherited metabolic disorder caused by ganglioside accumulation in the central nervous system, leading to severe and progressive neurological disorders (1). They are classified into GM1 and GM2-Gangliosidosis.

This study examined the clinical findings of 37 GM1&GM2-Gangliosidosis patients were referred to the neurology department of Mofid Children’s Hospital of SBUMs.

Approximately 40% of the patients were female, while 60% were male. Notably, 97.2% of the patients’ parents (36 cases) were related, suggesting a high prevalence of this disease in populations with consanguineous parentage. A separate 2014 study by the same center focused on 18 patients with GM2-Gangliosidosis, revealing that 83% of them were born to consanguineous parents.(14). 

The condition of the patients in terms of development in the four areas of gross motor, fine motor, communication and language, and social skill were evaluated retrospectively based on the ASQ3TM test before the onset of clinical symptoms of the disease. This study showed that the majority of juvenile GM1 and GM2-Gangliosidosis patients had reached their developmental milestones in the four areas of gross motor, fine motor, communication and Language, and social skills before the onset of disease symptoms. Only one GM1-Gangliosidosis patient was delayed in communication and language and one GM2-Gangliosidosis patient was in the gross motor area. In total, from 12 patients, 58% to 66% of them had developmental delay in different areas.

In the infantile GM2-Gangliosidosis, except in the gross motor area, which was more than 50% delayed, in other areas, more than 50% had reached their developmental milestone. This study shows that developmental delay in the patients of GM1-Gangliosidosis is more common than GM2-Gangliosidosis, but due to the small number of patients, it cannot be generalized, and more studies with a more significantnumber of patients are needed.

In general, in terms of development, all patients with GM1 and GM2-Gangliosidoses above 50% had reached their developmental milestones before the onset of disease symptoms.

In a retrospective study by Bley et al., 2011, they analyzed the neurological symptoms of ninety-two infantile patients out of 237 patients with GM2-Gangliosidosis. The study found that more than half of the infants achieved early motor developmental milestones, and those who did not achieve the early milestones did not achieve them later in life (15).

This study’s most the most common clinical symptoms observed in GM1 and GM2-Gangliosidosis patients included cherry-red spot, Mongolian spot, macrocephaly, organomegaly, hyperacusis, and scoliosis. Except for two patients of hyperacusis in juveniles, Tay-Sachs, and two patients of hepatomegaly in juveniles of Tay-Sachs and Sandhoff, none of the above symptoms were observed in juveniles of GM1 and GM2-Gangliosidosis. Almost all of our patients were regressed in movement and mental (except in one case). More than 75% of our patients had seizures during the disease, and the seizure form of most of them (64.3%) was mixed type and generalized tonic-clonic (14.3%).

In another study in 2014, at the same center, all 18 patients of GM2-Gangliosidosis had a developmental disorder as their main complaint. 38% of them had a history of developmental delay or regression, 22% had a history of seizures, and 88% of the patients had a cherry-red spot. After five years of investigation, all patients were hospitalized due to treatment-resistant seizures or aspiration pneumonia due to swallowing disorders, They concluded that the existence of cherry-red spot and hyperacusis, as well as seizures resistant to treatment in patients with related parents who have developmental delay or have regressed, are essential factors in patients suspected of GM2-Gangliosidosis (14).

In the brain MRI of twenty-four patients of this study, the most common finding reported was bilateral thalamus involvement in 6 (25%) infantile patients, as well as brain atrophy, PVL, delayed myelination, diffuse cerebellar atrophy, subdural space in fronto-parietal, extensive white matter signal change. Mild ventriculomegaly and hyperintensity in BG in T2 (lentiform nucleus) were other findings reported in the brain MRI of these patients. This study shows the more common involvement of the thalamus in these patients, along with other non-specific findings in their brain imaging.

In the study of 2014, the same center mentioned the most common imaging findings of GM2-Gangliosidosis patients, including bilateral involvement of the thalamus, brain atrophy, and hypomyelination in half of the patients (48%)(14).

In the review article by Andres et al., 2020 regarding Brain MRI findings of the Acute Infantile of GM2-Gangliosidosis, the most common finding was bilateral thalamic involvement, brain atrophy, and hypomyelination. Cerebellar atrophy is the most common finding in the juvenile subacute type, and severe cerebellar atrophy, hypodensity of the thalamus, is the most common finding in the adult chronic type (16).

In the electroencephalography of 12 patients with a history of seizures, four patients had normal EEG; three patients also had EEG with low voltage background; five patients had EEG with abnormal sharp waves.

In the study that Pampiglione et al., 1984 examined fifty-four GM1 and GM2-Gangliosidosis, they found that Irregular slow activity (Irregular slow activity) in low frequency and amplitude in most GM1 and GM2-Gangliosidosis patients is always abnormal. Since seizures occurred in most of our patients, perhaps paroxysmal features (spikes) are not a prominent feature in the EEG of GM2-Gangliosidosis or GM1 patients. When sharp waves or spikes were present, these waves were characteristically small in amplitude, usually mixed with slow activity, and showed a variable focal distribution (17).

## In Conclusion

This study showed that most of the patients result from family marriage. Most patients with juvenile GM1&GM2-Gangliosidosis are developmentally normal before the onset of the disease. Developmental delay is more common among infantile GM1-Gangliosidosis patients than infantile GM2-Gangliosidosis, but in total, more than 50% of GM1and GM2-Gangliosidosis patients reach their developmental milestone before the onset of the disease. More than 75% of patients suffer from seizures resistant to treatment during the disease. Common clinical findings in these patients are cherry-red spot, macrocephaly, hyperacusis, organomegaly, and Mongolian spot with bilateral involvement of the thalamus, and most of them experience motor and mental regression during the disease.

## Authorʼs Contribution

Dr. Karimzadeh P: Primary author (conception & design)

Ebrahimi M.: Secondary author and Corresponding author, Etemad K: statistical consultant, Ahmad Abadi: Academic advisor, Hoseininezhad Z: Academic advisor

## Conflict of Interest

The authors declare that they have no conflicts of interest regarding the research presented in this manuscript. 
